# De-escalation of antiplatelet therapy after percutaneous coronary intervention among East Asians and non-East Asians: a meta-analysis of randomized controlled trials

**DOI:** 10.1186/s12872-022-02476-1

**Published:** 2022-02-04

**Authors:** Ge Wang, Xiao Wang, Guanqi Zhao, Xin Huang, Ruifeng Guo, Shaoping Nie

**Affiliations:** Center for Coronary Artery Disease, Department of Cardiology, Beijing Anzhen Hospital, Capital Medical University, 2 Anzhen Road, Chaoyang District, Beijing, 100029 China

**Keywords:** Antiplatelet therapy, De-escalation, Meta-analysis, Percutaneous coronary intervention, Randomized controlled trials

## Abstract

**Background:**

To study the impact of de-escalation antiplatelet therapy retaining P2Y12 inhibition on major bleeding and ischemic outcomes after percutaneous coronary intervention (PCI) among East Asians and non-East Asians was unclear.

**Methods:**

We systematically searched PubMed, Embase, and the Cochrane Library for randomized controlled trials through September 2020. Eight trials were included, which studied de-escalation of DAPT (D-DAPT, switching to P2Y12 inhibitor monotherapy, or switching to clopidogrel or dose reduction of the P2Y12 inhibitor after 1 to 3 months) versus 12 months standard DAPT (S-DAPT). The primary outcomes data was conducted using random effects models.

**Results:**

Among the 8 included trials consisting of 37,775 patients, 62.6% presented with acute coronary syndrome. The median follow-up duration ranged from 12 to 24 months. Compared with S-DAPT, D-DAPT was associated with a lower risk of major bleeding (RR = 0.67, 95% CI 0.48–0.93, *p* = 0.02); however, this was only observed among East-Asians (RR = 0.61, 95% CI 0.37–0.99, *p* = 0.048). Among non-East Asians, the rate of major bleeding was similar between the two groups (RR = 0.73, 95% CI 0.46–1.14, *p* = 0.17, *p* for interaction = 0.59). There were no significant differences in the major adverse cardiovascular events (MACE) between D-DAPT and S-DAPT treatment among both East Asians (RR = 0.84, 95% CI 0.66–1.08, *p* = 0.18) and non-East Asians (RR = 0.89, 95% CI 0.79–1.00, *p* = 0.059*, p* for interaction = 0.71).

**Conclusions:**

The De-escalation strategy that retains P2Y12 inhibition after a PCI was associated with reduced risk of bleeding events, which was only demonstrated in East Asians patients, and not in non-East Asian patients.

**Supplementary Information:**

The online version contains supplementary material available at 10.1186/s12872-022-02476-1.

## Background

Currently, the American College of Cardiology (ACC), the American Heart Association (AHA), European, and Chinese guidelines all recommend that patients with acute coronary syndrome (ACS) undergoing percutaneous coronary interventions (PCI) receive 12 months of dual antiplatelet therapy (DAPT) and those with stable coronary artery diseases (SCAD) undergoing PCI should receive 6 months of DAPT as standard therapy (aspirin plus a P2Y12 inhibitor [1–6]). Given that most bleeding complications occur at 1 to 3 months after PCI, several large randomized controlled trials (RCTs) have evaluated the effects of de-escalation strategies [7–14]; however, the results are conflicting. Recently, several studies have reported that East Asian populations have different ischemic and bleeding profiles and might be more susceptible to bleeding [15–17]; thus, they may not benefit from more potent antithrombotic strategies [7–10]. Therefore, we conducted this meta-analysis to evaluate the impact of de-escalation strategies that retain the P2Y12 inhibitor in both bleeding and ischemic events among East Asian and non-East Asian populations. We present the following article in accordance with the PRISMA reporting checklist.

## Methods

### Data sources and search strategies

We searched PubMed, Embase, and the Cochrane Library for RCTs through September 2020 via two independent investigators (G. W. and X. W.) without language restrictions. The following keywords were used: “percutaneous coronary intervention” or “PCI” and “dual anti-platelet therapy” or “prasugrel” or “ticagrelor” or “clopidogrel” and “de-escalation” or “switch” or “monotherapy” and “Randomized Controlled Trials as Topic” (Additional file 2: Table S1–S3).

### Study selection and eligibility criteria

The two investigators (G. W. and X. W.) independently assessed studies considered for inclusion by screening the titles and abstracts. The inclusion criteria were: (1) RCTs that compared de-escalation of DAPT (D-DAPT, switching 1–3 months of DAPT to monotherapy with a P2Y12 receptor inhibitor, or switching to clopidogrel or dose reduction of the P2Y12 inhibitor) with standard DAPT (S-DAPT); (2) reported ischemic and bleeding events; (3) sample size > 500 patients. Studies that focused on duration of DAPT for ACS undergoing PCI or reported on the pharmacodynamics and pharmacokinetics of the drugs were excluded.

### Data extraction and quality assessment

Data extraction and adjudication were performed independently by two investigators (G. W. and X. W.) using a standardized electronic form. Any discrepancies were judged and solved by a senior author (S.N.). We recorded the following information: time span, region, demographic characteristics, the timing of de-escalation, switching strategies, number of events and participants, follow-up duration, and outcomes.

The potential risk of bias of the selected RCTs was assessed using the Cochrane Risk of Bias Tool [18]. This meta-analysis was performed according to the Preferred Reporting Items for Systematic Review and Meta-Analysis (PRISMA) statement [19].

### Outcome measures

The primary efficacy outcome was major adverse cardiovascular events (MACE), including all-cause or cardiovascular death, myocardial infarction, repeat revascularization, stent thrombosis, or stroke, which was reported by studies. The primary safety outcome was major bleeding, defined by the Bleeding Academic Research Consortium (BARC) ≥ 3, or Thrombolysis in Myocardial Infarction (TIMI) major bleeding. Secondary efficacy outcomes included cardiovascular death, all-cause death, myocardial infarction (MI), definite or probable stent thrombosis, repeat revascularization, and stroke. We compared the outcomes between two strategies among East Asian and non-East Asian populations according to ethnicity, all the included studies were also pooled into the analyses.

### Statistical analysis

A random effect model was used to evaluate all outcomes of interest among East Asians and non-East Asians patients, which were reported as the Risk Ratio (RR) with 95% confidence interval (CI). The Cochran Q test and I^2^ statistic were used to assess heterogeneity. The I^2^ statistic scores of 25%, 50%, and 75% represents low, moderate, and high heterogeneity, respectively. Sensitivity analyses were performed to evaluate the reasons for heterogeneity or the potential bias. Subgroup analyses of the primary outcomes were performed based on different de-escalation strategies to assess the safety and efficacy of different de-escalation strategies and locate the sources of heterogeneity. Forest plot was used to evaluate the overall effect of the trials, and egger’ test and funnel plots were used to assess publication bias. All data analysis was performed using Cochrane Review Manager software (version 5.4) and Stata 16. A 2-sided *p *value < 0.05 was considered statistically significant.

## Results

**Fig. 1:**
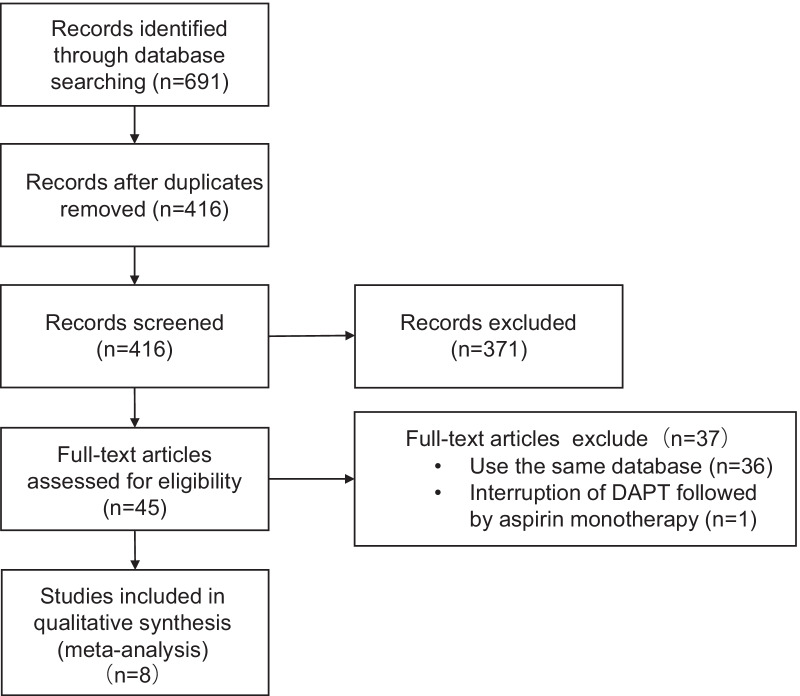
Flow chart of the study selection process of the meta-analysis

From 691 publications, 45 trials were assessed for full-text eligibility. After reading the full manuscripts, 37 studies were excluded, of which 36 studies were used same database. Finally, a total of 8 trials with 37,775 patients were included in this meta-analysis (Fig. 1). Overall, 23,637 patients (62.6%) presented as ACS, and the follow-up duration ranged from 12 to 24 months. Five trials evaluated P2Y12 inhibitor monotherapy compared with DAPT, two trials evaluated the switch from a potent P2Y12 inhibitor to clopidogrel, and one trial evaluated switching the dose of prasugrel from 10 to 5 mg. The detailed characteristics of all included studies are described in Table 1. The baseline clinical characteristics of each study are summarized in Table 2. The quality assessment is reported in Additional file 1: Figure S1. Among the 8 included studies, 87.5% had a high risk of blinding the intervention, and 100% had a low risk of blinding the outcome assessment.

**Table1:** Study design and clinical characteristics of included studies

Study name	Period	Reigon	Center	Population	D-DAPT	S-DAPT	Timing of de-escalation	Switching strategy	MACE	Bleeding	Follow-up time
HOST-REDUCE-POLYTECH-ACS	2014–2018	Korea	M	ACS	1170	1168	1 month after PCI	Prasugrel 10 mg–5 mg	Cardiac death + MI + stent thrombosis + ischemic stroke	BARC	1Y
TWILIGHT	2015–2017	North America + Europe + Asia	M	ACS + SCAD	3555	3564	3 months after discharge	Ticagrelor monotherapy	All-cause death + nonfatal MI + nonfatal stroke	BARC	15 mon
TICO	2015–2018	Korea	M	ACS	1527	1529	3-months after DAPT	Ticagrelor monotherapy	Death + MI + stent thrombosis + stroke + target-vessel revascularization	TIMI	1Y
TOPIC	2014–2016	France	S	ACS	323	323	1 month after ACS	Ticagrelor /prasugrel-clopidogrel	Cardiovascular death + unplanned urgent coronary revascularization + stroke	TIMI	1Y
TROPICAL-ACS	2013–2016	European	M	ACS	1304	1306	P(1-week) + c(1-week) and PFT-guided therapy from day 14	Prasugrel-clopidogrel (PFT-guided)	Cardiovascular death + MI + stroke	BARC	1Y
SMART CHOICE	2014–2018	Korea	M	SCAD + ACS	1495	1498	3-months after DAPT	A P2Y12 inhibitor monotherapy	All-cause mortality + MI + stroke	BARC	1Y
STOPDAPT-2	2015–2017	Japan	M	SCAD + ACS	1523	1522	1 month after DAPT	Clopidogrel monotherapy	Cardiovascular death + MI + definite stent thrombosis + stroke	BARC	1Y
GLOBAL-LEADERS	2013–2015	European	M	SCAD + ACS	7980	7988	1 month after DAPT	Ticagrelor monotherapy	All-cause mortality + stroke + new Q-wave MI	BARC	2Y

RCT, randomized controlled trials; ACS, acute coronary syndrome; SCAD, stable coronary artery disease; PCI, percutaneous coronary intervention; M, multi-center; S, single center; MACE, major adverse cardiovascular events; BARC, Bleeding Academic Research Consortium; TIMI, Thrombolysis In Myocardial Infraction major bleeding; Y, year; mon, month; P, prasugrel; C, clopidogrel; PFT, platelet function testing; DAPT, dual antiplatelet therapy; D-DAPT, de-escalation of dual antiplatelet therapy; S-DAPT, standard dual antiplatelet therapy; MI, myocardial infraction

**Table 2:** Baseline clinical characteristics of included studies

Study name	Age (y; m)	Female (n, %)	BMI (kg/m^2^; m)	DM (n, %)	Current smoker (n, %)	Prior MI (n, %)	Chronic kidney disease (n, %)	ACS (n, %)
HOST-REDUCE-POLYTECH-ACS [7]	58.8	252 (10.7)	25.8	990 (42.3)	838 (35.8)	90 (3.8)	64 (2.7)	2338 (100)
TWILIGHT [11]	65.2	1698 (23.9)	28.6	2620 (36.8)	1548 (21.8)	2040 (28.7)	1145 (16.8)	4614 (64.8)
TICO [9]	61	628 (20.5)	24.9	835 (27.3)	NA	113 (3.7)	620 (20.3)	3056 (100)
TOPIC [13]	60	114 (18)	27.2	177 (27)	286 (44)	NA	NA	646 (100)
TROPICAL-ACS [14]	58.8	2052 (78.6)	28.3	527 (20.2)	1182 (45.3)	293 (11.2)	67 (2.6)	2610 (100)
SMART CHOICE [8]	64.5	795 (26.6)	24.6	1122 (37.5)	791 (26.4)	127 (4.2)	97 (3.2)	1741 (58.2)
STOPDAPT-2 [10]	68.6	672 (22.3)	24.3	1159 (38.5)	710 (23.6)	406 (13.5)	166 (5.5)	1148 (38.2)
GLOBAL-LEADERS [12]	64.6	3714 (23.3)	28.2	4038 (25.3)	4169 (26.1)	3710 (23.2)	2171 (13.6)	7484 (46.9)

BMI, Body-mass index; DM, diabetes mellitus; MI, myocardial infraction; ACS, acute coronary syndrome; STEMI, ST-segment elevation myocardial infraction; y, years; m, mean; n, number; NA, not available

### Primary efficacy and safety outcomes

**Fig. 2:**
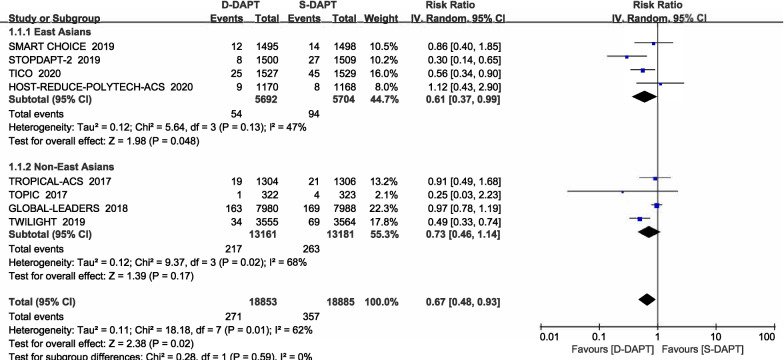
Forest plot of the risk estimates for major bleeding in patients treated with de-escalation using D-DAPT compared to S-DAPT

We analyzed the effect of D-DAPT on major bleeding in 8 trials. A total of 628 major bleeding events occurred in all participants. Treatment with D-DAPT was associated with a lower risk of major bleeding (RR = 0.67, 95% CI 0.48–0.93, *p* = 0.02), but this was only observed in studies including East Asians patients (0.9% vs. 1.6%, RR = 0.61, 95% CI 0.37–0.99, *p* = 0.048). Among non-East Asians, the rate of major bleeding was similar in the D-DAPT and S-DAPT groups (1.6% vs. 2.0%, RR = 0.73, 95% CI 0.46–1.14, *p* = 0.17). Furthermore, no interactions were observed between the East Asians and the non–East Asians (*p* for interaction = 0.59) (Fig. 2).

**Fig. 3:**
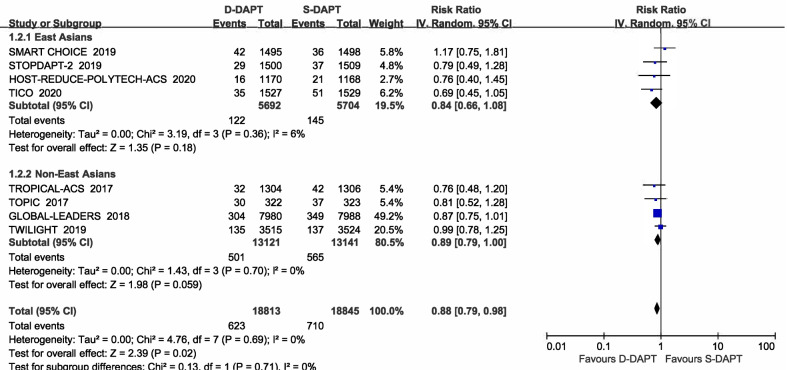
Forest plot of the risk estimates for MACE in patients treated with de-escalation of D-DAPT compared to S-DAPT

All trials reported the outcome of MACE. Treatment with de-escalation DAPT strategy was associated with a decreased risk of MACE (RR = 0.88, 95% CI 0.79–0.98, *p* = 0.02) in all participants. There was no significant difference of MACE between D-DAPT and S-DAPT treatment among both East Asians (RR = 0.84, 95% CI 0.66–1.08, *p* = 0.18) and non-East Asians (RR = 0.89, 95% CI 0.79–1.00, *p* = 0.059, *p* for interaction = 0.71) (Fig. 3).

### Cardiovascular death and all-cause death

**Fig. 4:**
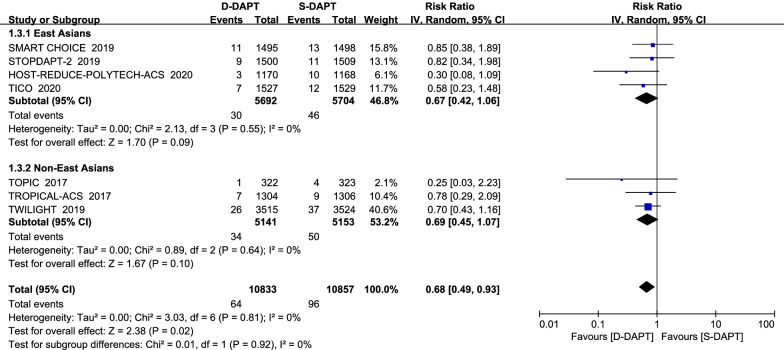
Forest plot of the risk estimates for cardiovascular death in patients treated with de-escalation of D-DAPT compared to S-DAPT

**Fig. 5:**
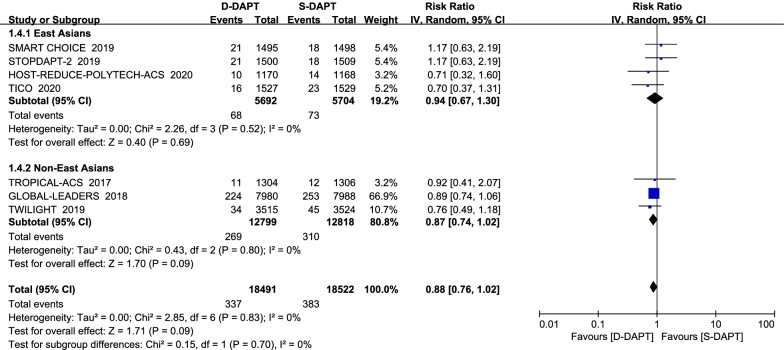
Forest plot of the risk estimates for all-cause death in patients treated with de-escalation of D-DAPT compared to S-DAPT

Among both the East Asians and the non-East Asians, there was no statistical significance in cardiovascular death (7 trials with 21,807 patients) between D-DAPT and S-DAPT groups (Fig. 4). Similarly, no statistical difference in all-cause death (7 trials with 37,129 patients) was observed in East Asians and non-East Asians (Fig. 5).

### Individual cardiovascular events

There were no statistical difference between D-DAPT and S-DAPT treatment concerning myocardial infarction (RR = 0.79, 95% CI 0.51–1.22, *p* = 0.29 in East Asians; RR = 0.99, 95% CI 0.86–1.14, *p* = 0.84 in non-East Asians, *p* for interaction = 0.35), definite or probable stent thrombosis (RR = 1.41, 95% CI 0.59–3.33, *p* = 0.44 in East Asians; RR = 0.93, 95% CI 0.69–1.26, *p* = 0.65 in non-East Asians, *p* for interaction = 0.38), repeat revascularization (RR = 1.15, 95% CI 0.91–1.46, *p* = 0.24 in East Asians; RR = 0.98, 95% CI 0.82–1.18, *p* = 0.84 in non-East Asians, *p* for interaction = 0.29), and stroke (RR = 0.95, 95% CI 0.52–1.77, *p* = 0.88 in East Asians; RR = 0.99, 95% CI 0.55–1.76, *p* = 0.97 in non-East Asians, *p* for interaction = 0.94) (Additional file 1: Figure S2 to Figure S5).

### Sensitivity analyses

There was a slight trend but no significant difference in MACE among non-East Asians between D-DAPT and S-DAPT groups. In contrast, the TROPICAL-ACS trial included a portion of patients who were re-escalated to prasugrel, which may affect the ischemic outcomes. After removing the TROPICAL-ACS trial, the risk of ischemic outcomes was similar among non-East Asians who received de-escalation of DAPT or standard DAPT. The risk of major bleeding events was significantly lower with de-escalation of DAPT in the initial pooled analysis; however, substantial heterogeneity was presented. After removing the GLOBAL-LEADERS trial, the risk of major bleeding was still lower with de-escalation of DAPT, while the heterogeneity I^2^ was reduced from 64 to 23%. This may be due to the large sample size of this trial. Among non-East Asians, the risk of major bleeding events was similar between the D-DAPT and S-DAPT groups. However, due to the unavailability of individual patients’ data in the TWILIGHT trial, which included one-sixth Asian, we pooled this trial into the subgroup of non-East Asians. After removing the TWILIGHT trial, the risk of major bleeding was still similar between the two groups among non-East Asians. Moreover, the de-escalation strategy used in the TOPIC trial and the TROPICAL-ACS trial is aspirin plus clopidogrel that is alike to the control group used in other trials (SMART CHOICE, GLOBAL LEADERS trial, STOP DAPT 2 trial) so that we performed a sensitivity analysis after removing these two trials finding consistency with the primary analysis (MACE: RR = 0.84, 95% CI 0.66–1.08, *p* = 0.18 in East Asians; RR = 0.90, 95% CI 0.80–1.03, *p* = 0.12 in Non-East Asians; Major bleeding: RR = 0.61, 95% CI 0.37–0.99, *p* = 0.048 in East Asians; RR = 0.71, 95% CI 0.37–1.36, *p* = 0.30 in Non-East Asians) (Additional file 1: Figure S7). Furthermore, we performed a subgroup analysis based on the de-escalation strategies in the overall population, only clopidogrel monotherapy strategy showed reduced major bleeding risks without increasing ischemic events (RR = 0.30, 95%CI = 0.14–0.65, Additional file 2: Table S4). There was no strong evidence of publication bias in funnel plots and egger’s regression tests for MACE and major bleeding (*p* = 0.55 and *p* = 0.079, respectively) (Additional file 1: Figure S6).

## Discussion

In the present meta-analysis, we found the following points: (1) Compared with S-DAPT, treatment with D-DAPT retaining a P2Y12 inhibitor was associated with lower risks of MACE and major bleeding complications; (2) Compared with S-DAPT, D-DAPT retaining a P2Y12 inhibitor was associated with a lower risk of major bleeding; however, this was only observed among East Asians. Among non-East Asians, the rate of major bleeding was similar between the two groups. (3) There were no significant associations between D-DAPT retaining a P2Y12 inhibitor treatment with cardiovascular death, all-cause death, and individual cardiovascular outcomes among East Asians and non-East Asians. Based on these results, compared with standard DAPT strategy, de-escalation of DAPT that retains P2Y12 inhibitor treatment might be considered an alternative DAPT strategy for East Asians.

It is well known that East Asians who received dual antiplatelet therapy after PCI have a higher risk of bleeding and a lower risk of ischemia [20]. The data is consistent with the results of our research. This may be due to the lower body mass index (BMI) of East Asians compared with non-East Asians. First, previous studies have shown that obesity is associated with thrombosis [21], and it is a prethrombotic state that could cause a series of changes in the body to promote the formation of a thrombus [22]. This may be one of the reasons why the risk of ischemia in East Asians is lower than that in non-East Asians. Second, the genetic polymorphism between different races may also explain the profile of East Asians. A previous Multi-Ethnic Study of Atherosclerosis (MESA) studies [23] have shown that African-Americans have the highest levels of dysfunctional endothelial profile (such as factor VIII, D-Dimer, plasmin–antiplasmin, and von Willebrand factor); therefore, they have the highest risk of thrombotic events, followed by Caucasians and Hispanics, and finally the Chinese. In addition, previous studies have shown that the level of inflammation can also affect thrombosis, and the level of inflammation in East Asians is lower than that in non-East Asians [24], which may also explain the low risk of ischemia in East Asians.

Previously, Bianco et al. (4 RCTs, 29,089 patients) indicated that after short-term DAPT followed by P2Y12 inhibitor monotherapy was associated with a lower risk of clinically relevant bleeding [odds ratio (OR) = 0.70, 95% CI 0.58–0.86] for patients undergoing PCI, as compared with 12 months DAPT, without an increased risk of 1-year cardiovascular events (OR = 0.90, 95% CI 0.79–1.03) [25]. Similar results were observed in the analysis by Michelle et al. (5 trials, 32,145 patients) [26]. Recently, Valgimigli et al. (6 RCTS, 24,096 patients) performed an individual patient level meta-analysis, they confirmed that P2Y12 inhibitor monotherapy was associated with a similar risk of ischemic events and lower rates of major bleeding compared with DAPT after coronary revascularization [27]. However, none of them included the latest research. Moreover, the population of these studies is vast, and they did not consider the differences in ethnicity. This present study extends prior studies by focusing on the role of short-term DAPT that retains a P2Y12 inhibitor therapy for the safety and efficacy outcomes among East Asians and non-East Asians. Our research confirmed that for East Asians, de-escalation strategy that retains the P2Y12 inhibitor was associated with a lower risk of major bleeding, but this was not observed among non-East Asians.

During the 1 to 3 months after stenting, which belongs to the high incidence phase of ischemic events after PCI, the thrombotic risk outweighs the bleeding risk. While bleeding events generally occurred during a longer period after stent implantation, which belongs to the chronic phase. From the studies we included, it was noted that most of the de-escalation strategies start at one or three months after receiving dual antiplatelet therapy. As mentioned before, East Asians have a profile of a high risk of bleeding. Therefore, in the chronic phase, downgrading dual antiplatelet therapy to reduce the degree of platelet inhibition might reduce the risk of bleeding events among East Asians [7–10]. This is consistent with the results of our meta-analysis. Among non-East Asians, we found that the effects of de-escalation of DAPT and standard DAPT on major bleeding events were similar and had no significant difference. However, the TWILIGHT study [12], which looked at both East-Asians and non-East Asians, showed that downgrading therapy could reduce bleeding events with a BARC ≥ 2 in Asians and Caucasians (HR = 0.49, 95CI%: 0.33–0.74). The study did not report the primary interest outcomes in East-Asians, and the effect on major bleeding events between East-Asians and non-East Asians was not clear. Furthermore, our sensitivity analysis showed that whether or not the study is removed, there is no significant effect on major bleeding outcomes of the non-East Asians subgroup. Therefore, the results of our meta-analysis can be considered reliable.

However, the results should be interpreted with caution. First, different definitions might influence the incidence of outcomes, including MACE and major bleeding events. Second, various de-escalation strategies among different races included in this analysis might affect the pooled analysis results. Finally, the TWILIGHT trial included both East-Asians and non-East Asians. Due to the lack of patient level data, we pooled all the data into the non-East Asians group.

### Limitations

This analysis has certain limitations. First, the rates of ischemic events were lower than anticipated in most of the included trials, resulting in limited statistical power for ischemia outcomes. Second, the included patients comprised of both ACS and SCAD, and due to lack of individual level data, we could not perform subgroup analysis for both groups. Third, adherence of agents was not noted, which was a recognized and common factor associated with long-term outcomes. Fourth, the race was judged by the sites of the participating studies. Therefore, the possibility of race mix-up was not excluded in those studies. However, since each patient category (East Asian versus other) depends on the study, the racial differences may be just differences among the studies. Fifth, the current analysis focused on major bleeding events, but minor bleeding was not reported, which more likely resulted in lower adherence to the treatment in clinical practice. Finally, although we strictly performed the study searching and selection, some potential publication or selection bias cannot be neglected.

## Conclusions

De-escalation strategy after PCI is associated with reduced risk of bleeding events, which was only demonstrated in East Asian patients, but not in non-East Asian patients. De-escalation of DAPT might be a safer and equally effective strategy for East Asians than the standard DAPT strategy.

## Supplementary Information

**Additional file 1: Figure S1.** Risk of bias summary for all included randomized controlled trials. **Figure S2.** Forest plot of the risk estimates for myocardial infraction in patients treated with de-escalation of D-DAPT compared to S-DAPT. **Figure S3.** Forest plot of the risk estimates for stent thrombosis in patients treated with de-escalation of D-DAPT compared to S-DAPT. **Figure S4.** Forest plot of the risk estimates for repeat revascularization in patients treated with de-escalation of D-DAPT compared to S-DAPT. **Figure S5.** Forest plot of the risk estimates for stroke in patients treated with de-escalation of D-DAPT compared to S-DAPT. **Figure S6.** Funnel plots of the included studies. A, funnel plot for major adverse cardiovascular events; B, funnel plot for major bleeding. **Figure S7a.** Forest plot of the risk estimates for MACE in patients treated with de-escalation of D-DAPT compared to S-DAPT for sensitivity analysis. **Figure S7b.** Forest plot of the risk estimates for Major bleeding in patients treated with de-escalation of D-DAPT compared to S-DAPT for sensitivity analysis.

**Additional file 2: Table S1.** PubMed. **Table S2.** Cochrane Library. **Table S3.** Embase **Table S4.** Subgroup analyses of primary outcomes between D-DAPT and S-DAPT strategies in overall populations

## Data Availability

Not applicable.

## Abbreviations

DAPT: Dual antiplatelet therapy
PCI: Percutaneous coronary intervention
D-DAPT: De-escalation of DAPT
S-DAPT: Standard DAPT
MACE: Major adverse cardiovascular event
RR: Risk Ratio
CI: Confidence intervals
ACC/AHA: American College of Cardiology/American Heart Association
ACS: Acute coronary syndrome
SCAD: Stable coronary artery diseases
RCTs: Randomized control trials
BMI: Body mass index
OR: Odds ratio

## References

[1] Collet JP, Thiele H, Barbato E, Barthelemy O, Bauersachs J, Bhatt DL, Dendale P, Dorobantu M, Edvardsen T, Folliguet T (2020). 2020 ESC Guidelines for the management of acute coronary syndromes in patients presenting without persistent ST-segment elevation. Eur Heart J.

[2] Levine GN, Bates ER, Bittl JA, Brindis RG, Fihn SD, Fleisher LA, Granger CB, Lange RA, Mack MJ, Mauri L (2016). 2016 ACC/AHA guideline focused update on duration of dual antiplatelet therapy in patients with coronary artery disease: a report of the American College of Cardiology/American Heart Association Task Force on Clinical Practice Guidelines. J Am Coll Cardiol.

[3] Valgimigli M, Byrne RA, Collet J-P, Costa F, Jeppsson A, Juni P (2018). 2017 ESC focused update on dual antiplatelet therapy in coronary artery disease developed in collaboration with EACTS. Eur Heart J.

[4] Junbo Ge YC (2017). Guideline and consensus for the management of patients with non-ST-elevation acute coronary syndrome (2016). Zhonghua Xin Xue Guan Bing Za Zhi.

[5] Ruiyan Zhang WG (2019). 2019 Chinese Society of Cardiology (CSC) guidelines for the diagnosis and management of patients with ST-segment elevation myocardial infarction. Zhonghua Xin Xue Guan Bing Za Zhi.

[6] Yanling Han WG, Fu X (2018). Guideline on the diagnosis and treatment of stable coronary artery disease. Zhonghua Xin Xue Guan Bing Za Zhi.

[7] Kim H-S, Kang J, Hwang D, Han J-K, Yang H-M, Kang H-J, Koo B-K, Rhew JY, Chun K-J, Lim Y-H (2020). Prasugrel-based de-escalation of dual antiplatelet therapy after percutaneous coronary intervention in patients with acute coronary syndrome (HOST-REDUCE-POLYTECH-ACS): an open-label, multicentre, non-inferiority randomised trial. The Lancet.

[8] Hahn JY, Song YB, Oh JH, Chun WJ, Park YH, Jang WJ, Im ES, Jeong JO, Cho BR, Oh SK (2019). Effect of P2Y12 inhibitor monotherapy vs dual antiplatelet therapy on cardiovascular events in patients undergoing percutaneous coronary intervention: the SMART-CHOICE randomized clinical trial. JAMA.

[9] Kim BK, Hong SJ, Cho YH, Yun KH, Kim YH, Suh Y, Cho JY, Her AY, Cho S, Jeon DW (2020). Effect of ticagrelor monotherapy vs ticagrelor with aspirin on major bleeding and cardiovascular events in patients with acute coronary syndrome: the TICO randomized clinical trial. JAMA.

[10] Watanabe H, Domei T, Morimoto T, Natsuaki M, Shiomi H, Toyota T, Ohya M, Suwa S, Takagi K, Nanasato M (2019). Effect of 1-month dual antiplatelet therapy followed by clopidogrel vs 12-month dual antiplatelet therapy on cardiovascular and bleeding events in patients receiving PCI: the STOPDAPT-2 randomized clinical trial. JAMA.

[11] Mehran R, Baber U, Sharma SK, Cohen DJ, Angiolillo DJ, Briguori C, Cha JY, Collier T, Dangas G, Dudek D (2019). Ticagrelor with or without aspirin in high-risk patients after PCI. N Engl J Med.

[12] Vranckx P, Valgimigli M, Jüni P, Hamm C, Steg PG, Heg D, van Es GA, McFadden EP, Onuma Y, van Meijeren C (2018). Ticagrelor plus aspirin for 1 month, followed by ticagrelor monotherapy for 23 months vs aspirin plus clopidogrel or ticagrelor for 12 months, followed by aspirin monotherapy for 12 months after implantation of a drug-eluting stent: a multicentre, open-label, randomised superiority trial. The Lancet.

[13] Cuisset T, Quilici J, Johnson TW, Deffarges S, Bassez C, Bonnet G, Fourcade L, Mouret JP, Lambert M, Verdier V, Morange PE, Alessi MC, Bonnet JL (2017). Benefit of switching dual antiplatelet therapy after acute coronary syndrome the TOPIC (timing of platelet inhibition after acute coronary syndrome) randomized study. Eur Heart J.

[14] Sibbing D, Aradi D, Jacobshagen C, Gross L, Trenk D, Geisler T, Orban M, Hadamitzky M, Merkely B, Kiss RG (2017). Guided de-escalation of antiplatelet treatment in patients with acute coronary syndrome undergoing percutaneous coronary intervention (TROPICAL-ACS): a randomised, open-label, multicentre trial. Lancet.

[15] Kang J, Park KW, Palmerini T, Stone GW, Lee MS, Colombo A, Chieffo A, Feres F, Abizaid A, Bhatt DL (2019). Racial differences in ischaemia/bleeding risk trade-off during anti-platelet therapy: individual patient level landmark meta-analysis from seven RCTs. Thromb Haemost.

[16] Numasawa Y, Sawano M, Fukuoka R, Ejiri K, Kuno T, Shoji S, Kohsaka S (2020). Antithrombotic strategy for patients with acute coronary syndrome: a perspective from East Asia. J Clin Med.

[17] Urban P, Mehran R, Colleran R, Angiolillo DJ, Byrne RA, Capodanno D, Cuisset T, Cutlip D, Eerdmans P, Eikelboom J (2019). Defining high bleeding risk in patients undergoing percutaneous coronary intervention: a consensus document from the Academic Research Consortium for High Bleeding Risk. Eur Heart J.

[18] Ghogomu EA, Maxwell LJ, Buchbinder R, Rader T, Pardo Pardo J, Johnston RV, Christensen RD, Rutjes AW, Winzenberg TM, Singh JA (2014). Updated method guidelines for cochrane musculoskeletal group systematic reviews and metaanalyses. J Rheumatol.

[19] Moher D, Clarke M, Ghersi D, Liberati A, Petticrew M, Shekelle P, Stewart LA (2015). Preferred reporting items for systematic review and meta-analysis protocols (PRISMA-P) 2015 statement. Syst Rev.

[20] Kang J, Kim HS (2018). The evolving concept of dual antiplatelet therapy after percutaneous coronary intervention: focus on unique feature of East Asian and "Asian Paradox". Korean Circ J.

[21] Samad F, Ruf W (2013). Inflammation, obesity, and thrombosis. Blood.

[22] Kornblith LZ, Howard B, Kunitake R, Redick B, Nelson M, Cohen MJ, Callcut R (2015). Obesity and clotting: body mass index independently contributes to hypercoagulability after injury. J Trauma Acute Care Surg.

[23] Lutsey PL, Cushman M, Steffen LM, Green D, Barr RG, Herrington D, Ouyang P, Folsom AR (2006). Plasma hemostatic factors and endothelial markers in four racial/ethnic groups: the MESA study. J Thromb Haemost.

[24] Veeranna V, Zalawadiya SK, Niraj A, Kumar A, Ference B, Afonso L (2013). Association of novel biomarkers with future cardiovascular events is influenced by ethnicity: results from a multi-ethnic cohort. Int J Cardiol.

[25] Bianco M, Careggio A, Destefanis P, Luciano A, Perrelli MG, Quadri G, Rossini R, Campo G, Vizzari G, D'Ascenzo F (2021). P2Y12 inhibitors monotherapy after short course of dual antiplatelet therapy in patients undergoing percutaneous coronary intervention: a meta-analysis of randomized clinical trials including 29 089 patients. Eur Heart J Cardiovasc Pharmacother.

[26] O’Donoghue ML, Murphy SA, Sabatine MS (2020). The safety and efficacy of aspirin discontinuation on a background of a P2Y12 inhibitor in patients after percutaneous coronary intervention: a systematic review and meta-analysis. Circulation.

[27] Valgimigli M, Gragnano F, Branca M, Franzone A, Baber U, Jang Y, Kimura T, Hahn JY, Zhao Q, Windecker S (2021). P2Y12 inhibitor monotherapy or dual antiplatelet therapy after coronary revascularisation: individual patient level meta-analysis of randomised controlled trials. BMJ.

