# Supplementary material for: De-escalation of antiplatelet therapy after percutaneous coronary intervention among East Asians and non-East Asians: a meta-analysis of randomized controlled trials
Source: BMC Cardiovasc Disord. 2022 Feb 4;22:29. doi: 10.1186/s12872-022-02476-1 (PMC8815247; doi:10.1186/s12872-022-02476-1)
Supplement: Supplementary file 1 — Additional file 1: Figure S1. Risk of bias summary for all included randomized controlled trials. Figure S2. Forest plot of the risk estimates for myocardial infraction in patients treated with de-escalation of D-DAPT compared to S-DAPT. Figure S3. Forest plot of the risk estimates for stent thrombosis in patients treated with de-escalation of D-DAPT compared to S-DAPT. Figure S4. Forest plot of the risk estimates for repeat revascularization in patients treated with de-escalation of D-DAPT compared to S-DAPT. Figure S5. Forest plot of the risk estimates for stroke in patients treated with de-escalation of D-DAPT compared to S-DAPT. Figure S6. Funnel plots of the included studies. A, funnel plot for major adverse cardiovascular events; B, funnel plot for major bleeding. Figure S7a. Forest plot of the risk estimates for MACE in patients treated with de-escalation of D-DAPT compared to S-DAPT for sensitivity analysis. Figure S7b. Forest plot of the risk estimates for Major bleeding in patients treated with de-escalation of D-DAPT compared to S-DAPT for sensitivity analysis. [file 12872_2022_2476_MOESM1_ESM.pdf]

PRISMA 2009 Checklist

Section/topic	Item No	Checklist item	Reported on Page Number/Line Number	Reported on Section/Paragraph
**TITLE**				
Title	1	Identify the report as a systematic review, meta-analysis, or both.	1	title
**ABSTRACT**				
Structured summary	2	Provide a structured summary including, as applicable: background; objectives; data sources; study eligibility criteria, participants, and interventions; study appraisal and synthesis methods; results; limitations; conclusions and implications of key findings; systematic review registration number.	2-3	Abstract and keywords
**INTRODUCTION**				
Rationale	3	Describe the rationale for the review in the context of what is already known.	4	Backgoound
Objectives	4	Provide an explicit statement of questions being addressed with reference to participants, interventions, comparisons, outcomes, and study design (PICOS).	4	Background
**METHODS**				
Protocol and registration	5	Indicate if a review protocol exists, if and where it can be accessed (e.g., Web address), and, if available, provide registration information including registration number.		
Eligibility criteria	6	Specify study characteristics (e.g., PICOS, length of follow-up) and report characteristics (e.g., years considered, language, publication status) used as criteria for eligibility, giving rationale.	5	Study Selection and Eligibility Criteria
Information sources	7	Describe all information sources (e.g., databases with dates of coverage, contact with study authors to identify additional studies) in the search and date last searched.	4-5	Data Sources and Search Strategies
Search	8	Present full electronic search strategy for at least one database, including any limits used, such that it could be repeated.	4-5	Data Sources and Search Strategies
Study selection	9	State the process for selecting studies (i.e., screening, eligibility, included in systematic review, and, if applicable, included in the meta-analysis).	5	Study Selection and Eligibility Criteria
Data collection process	10	Describe method of data extraction from reports (e.g., piloted forms, independently, in duplicate) and any processes for obtaining and confirming data from investigators.	5-6	Data Extraction and Quality Assessment
Data items	11	List and define all variables for which data were sought (e.g., PICOS, funding sources) and any assumptions and simplifications made.	5-6	Data Extraction and Quality Assessment

Risk of bias in individual studies	12	Describe methods used for assessing risk of bias of individual studies (including specification of whether this was done at the study or outcome level), and how this information is to be used in any data synthesis.	6	Data Extraction and Quality Assessment
Summary measures	13	State the principal summary measures (e.g., risk ratio, difference in means).	6	Outcome Measures
Synthesis of results	14	Describe the methods of handling data and combining results of studies, if done, including measures of consistency (e.g., I^2) for each meta-analysis.	6-7	Statistical Analysis
Risk of bias across studies	15	Specify any assessment of risk of bias that may affect the cumulative evidence (e.g., publication bias, selective reporting within studies).	6	Data Extraction and Quality Assessment
Additional analyses	16	Describe methods of additional analyses (e.g., sensitivity or subgroup analyses, meta-regression), if done, indicating which were pre-specified.	6-7	Statistical Analysis
**RESULTS**				
Study selection	17	Give numbers of studies screened, assessed for eligibility, and included in the review, with reasons for exclusions at each stage, ideally with a flow diagram.	7	Results/1
Study characteristics	18	For each study, present characteristics for which data were extracted (e.g., study size, PICOS, follow-up period) and provide the citations.	7	Results/1
Risk of bias within studies	19	Present data on risk of bias of each study and, if available, any outcome level assessment (see item 12).	7	Results/1
Results of individual studies	20	For all outcomes considered (benefits or harms), present, for each study: (a) simple summary data for each intervention group (b) effect estimates and confidence intervals, ideally with a forest plot.	8-9	1-4
Synthesis of results	21	Present results of each meta-analysis done, including confidence intervals and measures of consistency.	8-9	1-4
Risk of bias across studies	22	Present results of any assessment of risk of bias across studies (see Item 15).	7	Results/1
Additional analysis	23	Give results of additional analyses, if done (e.g., sensitivity or subgroup analyses, meta-regression [see Item 16]).	9-10	Sensitivity Analyses
**DISCUSSION**				
Summary of evidence	24	Summarize the main findings including the strength of evidence for each main outcome; consider their relevance to key groups (e.g., healthcare providers, users, and policy makers).	10-13	1-5
Limitations	25	Discuss limitations at study and outcome level (e.g., risk of bias), and at review-level (e.g., incomplete retrieval of identified research, reporting bias).	14	Limitations:
Conclusions	26	Provide a general interpretation of the results in the context of other evidence, and implications for future research.	14	Conclusions

FUNDING				
Funding	27	Describe sources of funding for the systematic review and other support (e.g., supply of data); role of funders for the systematic review.	15	Acknowledgments

From: Moher D, Liberati A, Tetzlaff J, Altman DG, The PRISMA Group (2009). Preferred Reporting Items for Systematic Reviews and Meta-Analyses: The PRISMA Statement. PLoS Med 6(7): e1000097. doi:10.1371/journal.pmed1000097

For more information, visit: www.prisma-statement.org.

Please leave this space alone as it will be supplemented by the editorial office when needed.

Supplemental Figures

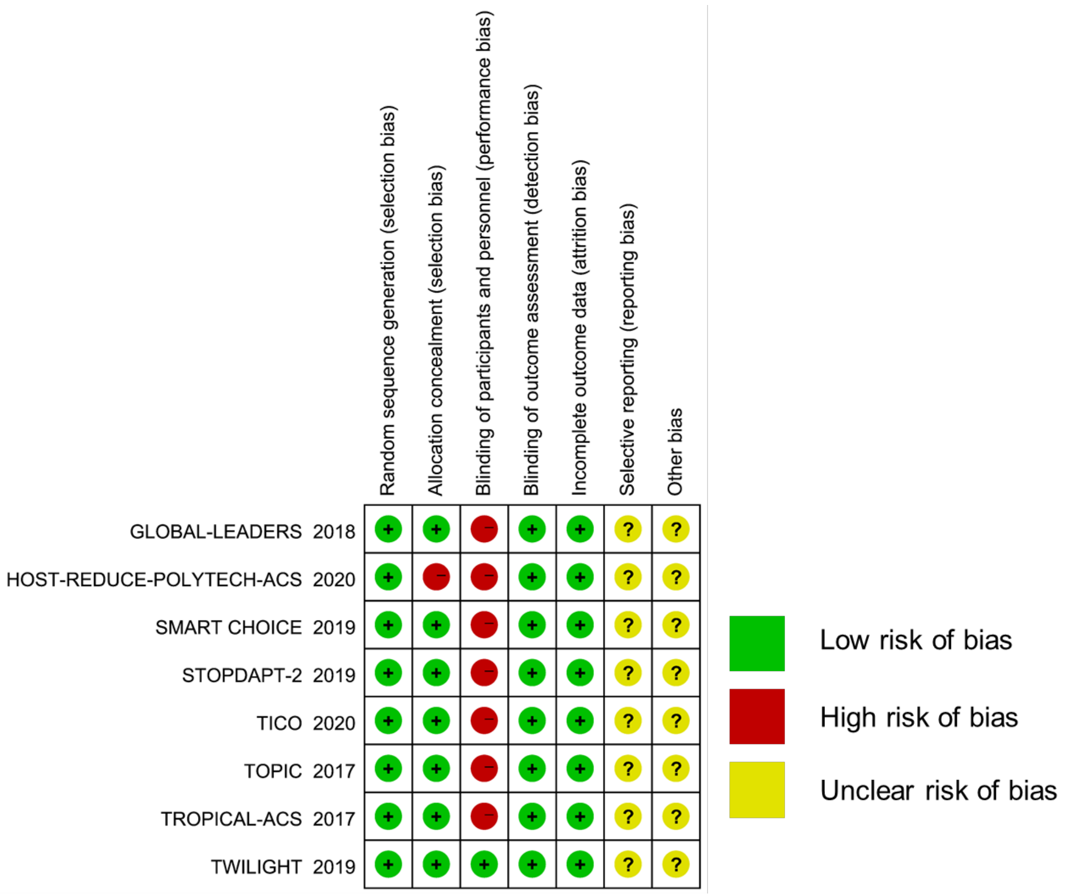

Figure S1. Risk of bias summary for all included randomized controlled trials

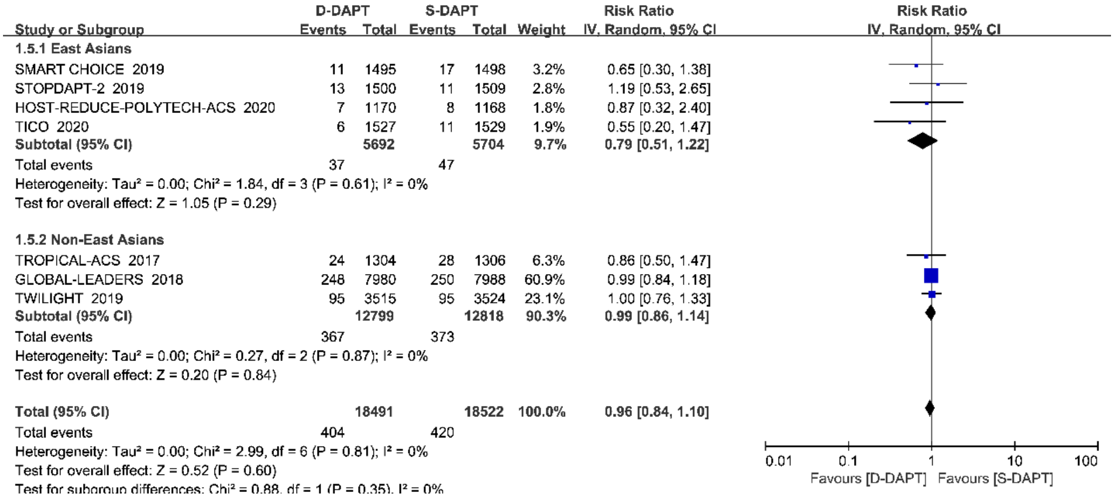

Figure S2. Forest plot of the risk estimates for myocardial infraction in patients treated with de-escalation of D-DAPT compared to S-DAPT

Study or Subgroup	D-DAPT Events	D-DAPT Total	S-DAPT Events	S-DAPT Total	Weight	Risk Ratio IV, Random, 95% CI
**1.6.1 East Asians**						
SMART CHOICE 2019	3	1495	2	1498	2.6%	1.50 [0.25, 8.98]
STOPDAPT-2 2019	4	1500	1	1509	1.7%	4.02 [0.45, 35.96]
HOST-REDUCE-POLYTECH-ACS 2020	1	1170	3	1168	1.6%	0.33 [0.03, 3.19]
TICO 2020	6	1527	4	1529	5.2%	1.50 [0.42, 5.31]
**Subtotal (95% CI)**		**5692**		**5704**	**11.0%**	**1.41 [0.59, 3.33]**
Total events	14		10			
Heterogeneity: Tau² = 0.00; Chi² = 2.46, df = 3 (P = 0.48); I² = 0%						
Test for overall effect: Z = 0.77 (P = 0.44)						
**1.6.2 Non-East Asians**						
TROPICAL-ACS 2017	2	1304	3	1306	2.6%	0.67 [0.11, 3.99]
GLOBAL-LEADERS 2018	64	7980	64	7988	69.0%	1.00 [0.71, 1.41]
TWILIGHT 2019	14	3515	19	3524	17.3%	0.74 [0.37, 1.47]
**Subtotal (95% CI)**		**12799**		**12818**	**89.0%**	**0.93 [0.69, 1.26]**
Total events	80		86			
Heterogeneity: Tau² = 0.00; Chi² = 0.74, df = 2 (P = 0.69); I² = 0%						
Test for overall effect: Z = 0.45 (P = 0.65)						
**Total (95% CI)**		**18491**		**18522**	**100.0%**	**0.98 [0.73, 1.30]**
Total events	94		96			
Heterogeneity: Tau² = 0.00; Chi² = 3.97, df = 6 (P = 0.68); I² = 0%						
Test for overall effect: Z = 0.17 (P = 0.87)						
Test for subgroup differences: Chi² = 0.77, df = 1 (P = 0.38), I² = 0%						

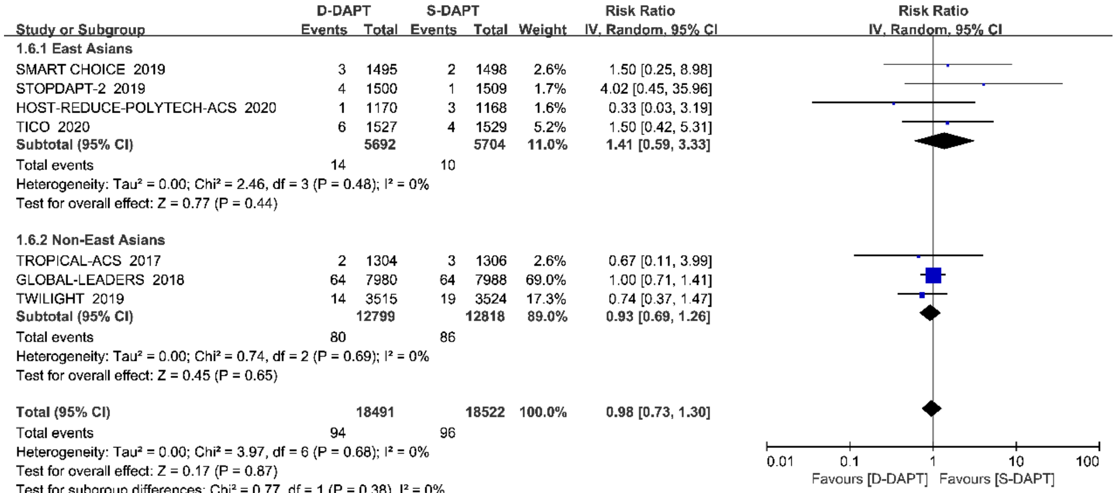

Figure S3. Forest plot of the risk estimates for stent thrombosis in patients treated with de-escalation of D-DAPT compared to S-DAPT

Study or Subgroup	D-DAPT Events	D-DAPT Total	S-DAPT Events	S-DAPT Total	Weight	Risk Ratio IV, Random, 95% CI
**1.7.1 East Asians**						
STOPDAPT-2 2019	98	1500	76	1509	19.6%	1.30 [0.97, 1.74]
HOST-REDUCE-POLYTECH-ACS 2020	35	1170	37	1168	9.9%	0.94 [0.60, 1.49]
TICO 2020	8	1527	10	1529	2.7%	0.80 [0.32, 2.02]
**Subtotal (95% CI)**		**4197**		**4206**	**32.1%**	**1.15 [0.91, 1.46]**
Total events	141		123			
Heterogeneity: Tau² = 0.00; Chi² = 1.96, df = 2 (P = 0.37); I² = 0%						
Test for overall effect: Z = 1.18 (P = 0.24)						
**1.7.2 Non-East Asians**						
TOPIC 2017	28	322	30	323	8.7%	0.94 [0.57, 1.53]
TROPICAL-ACS 2017	40	1304	29	1306	9.3%	1.38 [0.86, 2.21]
GLOBAL-LEADERS 2018	739	7980	793	7988	49.9%	0.93 [0.85, 1.03]
**Subtotal (95% CI)**		**9606**		**9617**	**67.9%**	**0.98 [0.82, 1.18]**
Total events	807		852			
Heterogeneity: Tau² = 0.01; Chi² = 2.56, df = 2 (P = 0.28); I² = 22%						
Test for overall effect: Z = 0.21 (P = 0.84)						
**Total (95% CI)**		**13803**		**13823**	**100.0%**	**1.03 [0.88, 1.20]**
Total events	948		975			
Heterogeneity: Tau² = 0.01; Chi² = 6.83, df = 5 (P = 0.23); I² = 27%						
Test for overall effect: Z = 0.36 (P = 0.72)						
Test for subgroup differences: Chi² = 1.13, df = 1 (P = 0.29), I² = 11.7%						

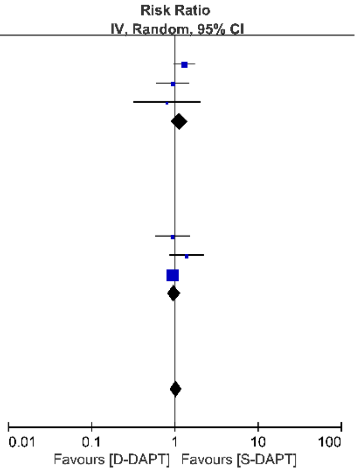

Figure S4. Forest plot of the risk estimates for repeat revascularization in patients treated with de-escalation of D-DAPT compared to S-DAPT

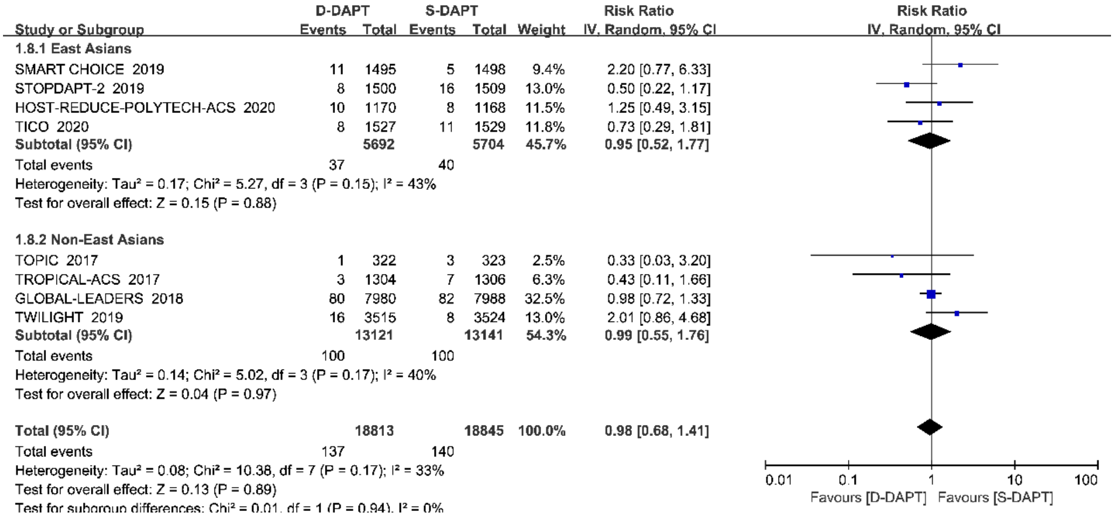

Figure S5. Forest plot of the risk estimates for stroke in patients treated with de-escalation of D-DAPT compared to S-DAPT

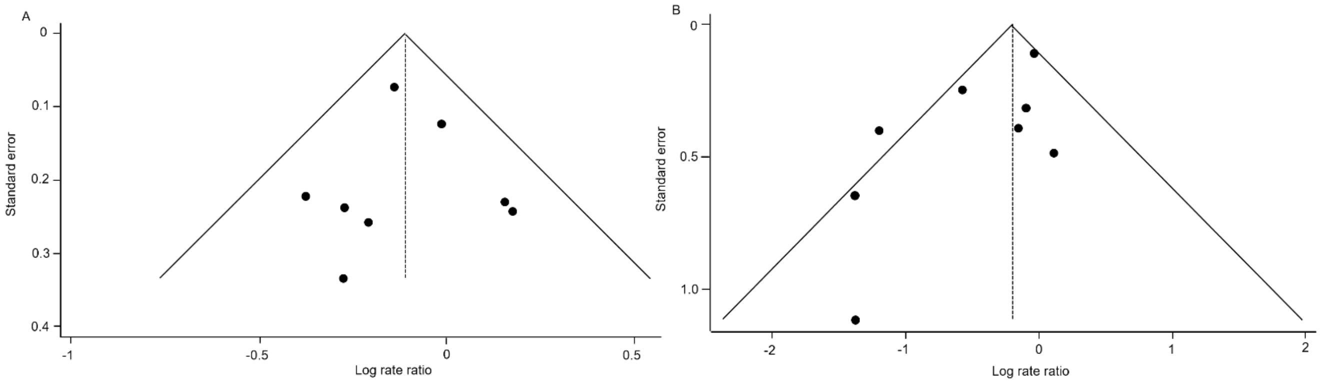

Figure S6. Funnel plots of the included studies. A, funnel plot for major adverse cardiovascular events; B, funnel plot for major bleeding.

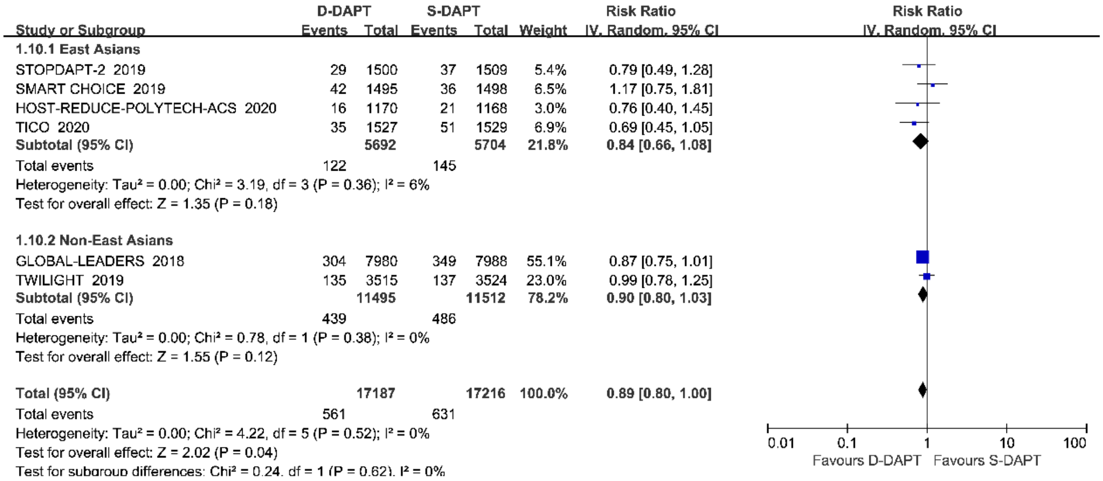

Figure S7a. Forest plot of the risk estimates for MACE in patients treated with de-escalation of D-DAPT compared to S-DAPT for sensitivity analysis

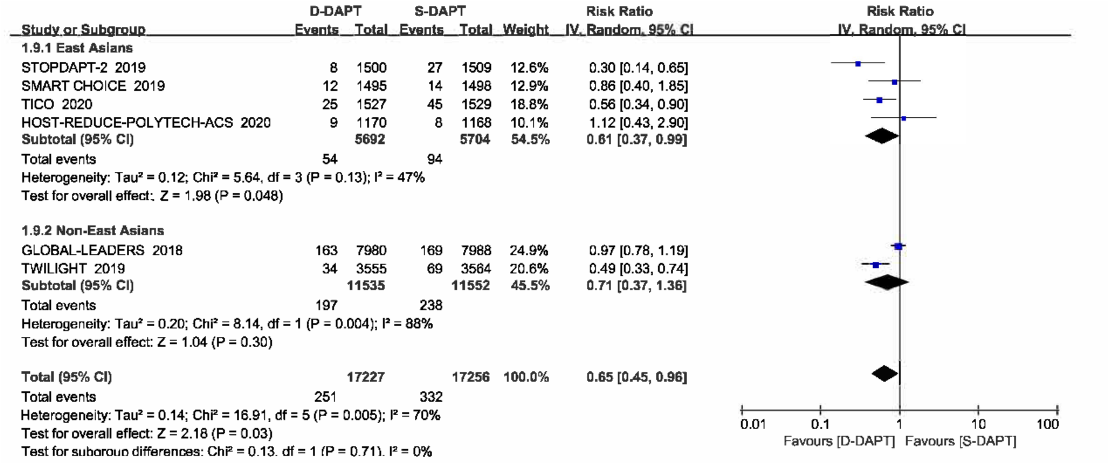

Figure S7b. Forest plot of the risk estimates for Major bleeding in patients treated with de-escalation of D-DAPT compared to S-DAPT for sensitivity analysis
