# Supplementary material for: De-escalation of antiplatelet therapy after percutaneous coronary intervention among East Asians and non-East Asians: a meta-analysis of randomized controlled trials
Source: BMC Cardiovasc Disord. 2022 Feb 4;22:29. doi: 10.1186/s12872-022-02476-1 (PMC8815247; doi:10.1186/s12872-022-02476-1)
Supplement: Supplementary file 2 — Additional file 2: Table S1. PubMed. Table S2. Cochrane Library. Table S3. Embase Table S4. Subgroup analyses of primary outcomes between D-DAPT and S-DAPT strategies in overall populations [file 12872_2022_2476_MOESM2_ESM.docx]

**Search Strategy**

**Table S1. PubMed**

| **#** | **Search history** | **Records** |
| --- | --- | --- |
| 1 | “Percutaneous Coronary Intervention” [MeSH] | 59654 |
| 2 | percutaneous[tw] AND coronary[tw] AND intervention[tw] | 48259 |
| 3 | PCI [tw] | 30093 |
| 4 | coronary[tw] AND (intervention*[tw]OR revascularization*[tw]OR angioplast*[tw]) | 135048 |
| 5 | coronary[tw] AND Stent*[tw] | 43437 |
| 6 | #1 OR #2 OR #3 OR #4 OR #5 | 149662 |
| 7 | “Dual Anti-Platelet Therapy” [MeSH] | 408 |
| 8 | “Platelet Aggregation Inhibitors” [MeSH] | 38906 |
| 9 | “Purinergic p2y Receptor Antagonists” [MeSH] OR Thienopyridines [MeSH] OR “Thromboxane A2” [MeSH] | 18645 |
| 10 | dual antiplatelet[tw] OR antiplatelet*[tw] OR anti-platelet*[tw] | 35611 |
| 11 | aspirin[tw] OR clopidogrel[tw] OR ticagrelor[tw] OR prasugrel[tw] | 79094 |
| 12 | #7 OR #8 OR #9 OR#10 OR #11 | 119195 |
| 13 | switch[tw]) OR de-escalation[tw] OR downgrade[tw] OR monotherapy[tw] | 141066 |
| 14 | “Randomized Controlled Trials as Topic” [MeSH] | 154829 |
| 15 | randomized[tw] | 993176 |
| 16 | randomly[tw] | 372698 |
| 17 | placebo[tw] | 231194 |
| 18 | clinical trials[tw] | 459760 |
| 19 | #14 OR #15 OR #16 OR#17 OR #18 | 1556068 |
| 20 | animals[MeSH]) NOT humans[MeSH] | 4930424 |
| 21 | #19 NOT #20 | 1437647 |
| 22 | "1996/01/01"[Date - Publication] : "2020/09/01"[Date - Publication] | 19944849 |
| 20 | #6 AND #12 AND #13 AND #21 AND #22 | 195 |

**Table S2. Cochrane Library**

| **#** | **Search history** | **Records** |
| --- | --- | --- |
| 1 | [mh “Percutaneous Coronary Intervention”] | 2401 |
| 2 | (percutaneous coronary near/2 (interven* or revascular*)):ti,ab,kw | 11302 |
| 3 | PCI:ti,ab,kw | 9224 |
| 4 | (coronary stent*):ti,ab,kw | 9059 |
| 5 | (coronary near/3 angioplast*):ti,ab,kw | 5427 |
| 6 | #1 or #2 or #3 or #4 or #5 | 19909 |
| 7 | [mh “Dual Anti-Platelet Therapy”] | 56 |
| 8 | [mh “Platelet Aggregation Inhibitors”] | 4112 |
| 9 | [mh “Purinergic p2y Receptor Antagonists”] or [mh Thienopyridines] or [mh “Thromboxane A2”] | 558 |
| 10 | (dual antiplatelet or antiplatelet*or anti-platelet*):ti,ab,kw | 2198 |
| 11 | (Aspirin or clopidogrel or ticagrelor or prasugrel):ti,ab,kw | 18234 |
| 12 | #7 or #8 or #9 or #10 or #11 | 20186 |
| 13 | (switch or de-escalation or downgrade or monotherapy):ti,ab,kw | 32892 |
| 14 | [mh “Randomized Controlled Trials as Topic”] | 12755 |
| 15 | randomized:ti,ab,kw | 987785 |
| 16 | randomly:ti,ab,kw | 272555 |
| 17 | placebo:ti,ab,kw | 328307 |
| 18 | clinical trials:ti,ab,kw | 171694 |
| 19 | #14 or #15 or #16 or #17 or #18 | 1209342 |
| 20 | [mh“Animals”] not [mh “Humans”] | 4 |
| 21 | #19 not #20 | 1209339 |
| 22 | #6 and #12 and #13 and #21 | 275 |
| 23 | “#23” with Cochrane Library publication date Between Jan 1996 and Sep 2020 | 214 |

**Table S3. Embase**

| **#** | **Search history** | **Records** |
| --- | --- | --- |
| 1 | 'percutaneous coronary intervention'/exp | 113045 |
| 2 | percutaneous:ab,ti AND ((coronary NEAR/2 (interven* OR revascular*)):ab,ti) | 67037 |
| 3 | pci:ab,ti | 64497 |
| 4 | coronary:ab,ti AND artery:ab,ti AND stent*:ab,ti | 28336 |
| 5 | (coronary NEAR/3 angioplast*):ab,ti | 18727 |
| 6 | #1 or #2 or #3 or #4 or #5 | 159135 |
| 7 | 'dual antiplatelet therapy'/exp | 9937 |
| 8 | 'antithrombocytic agent'/exp | 377798 |
| 9 | 'purinergic P2Y receptor antagonist'/exp OR 'thienopyridine derivative'/exp OR 'thromboxane A2'/exp | 89388 |
| 10 | 'dual antiplatelet':ab,ti OR antiplatelet*:ab,ti OR 'anti platelet*':ab,ti | 59043 |
| 11 | aspirin:ab,ti OR clopidogrel:ab,ti OR ticagrelor:ab,ti OR prasugrel:ab,ti | 94432 |
| 12 | #7 or #8 or #9 or #10 or #11 | 410545 |
| 13 | switch:ab,ti OR 'de-escalation':ab,ti OR downgrade:ab,ti OR monotherapy:ab,ti | 209479 |
| 14 | 'randomized controlled trial (topic)'/exp | 216250 |
| 15 | randomized:ab | 785953 |
| 16 | randomly:ab | 491772 |
| 17 | placebo:ab | 324753 |
| 18 | 'clinical trials':ab | 374097 |
| 19 | #14 OR #15 OR #16 OR#17 OR #18 | 1751607 |
| 20 | 'animal'/exp NOT 'human'/exp | 5702708 |
| 21 | #19 NOT #20 | 1578126 |
| 22 | [1996-2020]/py | 25979709 |
| 23 | #6 AND #12 AND #13 AND #21 AND #22 | 282 |

| Table S4. Subgroup analyses of primary outcomes between D-DAPT and S-DAPT strategies in overall populations | | | | | | | | | | |
| --- | --- | --- | --- | --- | --- | --- | --- | --- | --- | --- |
| Subgroups | Study no. | D-DAPT | |  | S-DAPT | | Risk ratio (95%CI) | Heterogeneity | | *P* _for interaction_ |
|  |  | Events | Subjects |  | Events | Subjects |  | *I^2^* | *p* value |  |
| Major bleeding | | | | | | | | | | |
| Different de-escalation strategy | | | | | | | | | | |
| Ticagrelor monotherapy | 3 | 222 | 13062 |  | 283 | 13081 | 0.66 (0.41, 1.07) | 81% | 0.005 | 0.17 |
| Clopidogrel monotherapy | 1 | 8 | 1500 |  | 277 | 1509 | 0.30 (0.14, 0.65) | NA | NA |  |
| Lower dose prasugrel | 2 | 20 | 1626 |  | 25 | 1629 | 0.74 (0.30, 1.85) | 19% | 0.27 |  |
| Switch to clopidogrel | 1 | 9 | 1170 |  | 8 | 1168 | 1.12 (0.43, 2.90) | NA | NA |  |
| MACE | | | | | | | | | | |
| Different de-escalation strategy | | | | | | | | | | |
| Ticagrelor monotherapy | 3 | 474 | 13031 |  | 537 | 13032 | 0.88 (0.77, 1.01) | 8% | 0.34 | 0.87 |
| Clopidogrel monotherapy | 1 | 29 | 1500 |  | 37 | 1509 | 0.79 (0.49, 1.28) | NA | NA |  |
| Lower dose prasugrel | 2 | 62 | 1626 |  | 79 | 1629 | 0.79 (0.57, 1.09) | 0% | 0.85 |  |
| Switch to clopidogrel | 1 | 16 | 1170 |  | 21 | 1168 | 0.76 (0.40, 1.45) | NA | NA |  |
| DAPT: dual antiplatelet therapy; D-DAPT: de-escalation of DAPT; S-DAPT: standard DAPT; CI: confidence interval; no.: number; MACE: major adverse cardiovascular events. | | | | | | | | | | |
